# Real‐life effectiveness of biological therapies on symptoms in severe asthma with comorbid CRSwNP

**DOI:** 10.1002/clt2.12049

**Published:** 2021-07-30

**Authors:** Carlo Mümmler, Kristin Dünzelmann, Nikolaus Kneidinger, Michaela Barnikel, Dieter Munker, Moritz Gröger, Martin Canis, Jürgen Behr, Andrea Koch, Frank Haubner, Katrin Milger

**Affiliations:** ^1^ Department of Medicine V University Hospital LMU Munich Munich Germany; ^2^ Comprehensive Pneumology Center (CPC‐M) LMU and Helmholtz Center Munich Munich Germany; ^3^ Department of Otorhinolaryngology University Hospital LMU Munich Munich Germany; ^4^ Abteilung für Lungenheilkunde Pyhrn‐Eisenwurzen Klinikum Steyr Munich Austria

**Keywords:** antibody, asthma, benralizumab, biological, CRSwNP, dupilumab, mepolizumab, nasal polyps, omalizumab, real‐life

## Abstract

**Background:**

We aimed to evaluate the effectiveness of different antibody therapies on nasal polyp symptoms in patients treated for severe asthma.

**Methods:**

We performed a retrospective analysis of patients with severe asthma and comorbid CRSwNP who were treated with anti‐IgE, anti‐IL‐5/R or anti‐IL‐4R. CRSwNP symptom burden was evaluated before and after 6 months of therapy.

**Results:**

Fifty patients were included hereof treated with anti‐IgE: 9, anti‐IL‐5/R: 26 and anti‐IL‐4R: 15 patients. At baseline median SNOT‐20 was similar among groups (anti‐IgE: 55, anti‐IL‐5/R: 52 and anti‐IL‐4R: 56, *p* = 0.76), median visual analogue scale (VAS) for nasal symptoms was 4, 7 and 8 (*p* = 0.14) and VAS for total symptoms was higher in the anti‐IL‐4R group (4, 5 and 8, *p* = 0.002). After 6 months SNOT‐20 improved significantly in all patient groups with median improvement of anti‐IgE: −8 (*p* < 0.01), anti‐IL‐5/R: −13 (*p* < 0.001) and anti‐IL‐4R: −18 (*p* < 0.001), with larger improvement in the anti‐IL‐4R group than in anti‐IgE (*p* < 0.001) and anti‐IL‐5/R (*p* < 0.001) groups. VAS nasal symptoms improved by median anti‐IgE: 0 (n.s.), anti‐IL‐5/R: −1 (*p* < 0.01) and anti‐IL‐4R: −3 (*p* < 0.001), VAS total symptoms by anti‐IgE: −1 (n.s.), anti‐IL‐5/R: −2 (*p* < 0.001) and anti‐IL‐4R: −2 (*p* < 0.001).

**Conclusions:**

Treatment by all antibodies showed effectiveness in reducing symptoms of CRSwNP in patients with severe asthma, with the largest reduction observed in anti‐IL‐4R‐treated patients.

## TO THE EDITOR

Chronic rhinosinusitis with nasal polyps (CRSwNP) is a frequent comorbidity in severe asthma^1^ and shares key pathophysiological mechanisms including increased type 2‐inflammation with secretion of IL‐5, IL‐4, IL‐13 and IgE. Biological therapies, namely IgE‐, IL‐5/IL‐5Rα ‐ and IL‐4Rα ‐ antibodies have revolutionized the treatment of severe asthma and were recently also found to be effective in severe CRSwNP.^2^, ^3^, ^4^ Here, we investigated the real‐life effectiveness of different biologics on symptoms in patients with severe asthma and comorbid CRSwNP.

We performed a retrospective analysis of patients from LMU Munich severe asthma cohort included in the prospective German Asthma Net (GAN) registry who fulfilled inclusion criteria. The registry was approved by the IRB and all patients provided written informed consent. All patients fulfilled the diagnosis of severe asthma according to ERS/ATS guidelines.^5^ Inclusion criteria: Patients with severe asthma and comorbid CRSwNP, who were initiated with monoclonal antibody therapy between 2018 and 2020 for whom data at baseline (−4 to 0 weeks before first antibody injection) and after 6 months (+/− 4 weeks) of antibody therapy were available. Current CRSwNP was defined as either a confirmed diagnosis by an ENT specialist, or typical nasal symptoms together with a CT scan evidencing nasal polyps and regular use of mometasone intranasally throughout the study period. History of previous nasal polyp operations was self‐reported. All patients received high‐dose ICS/LABA +/− LAMA throughout the study, and some additionally oral corticosteroids (OCS, Table 1). Outcome measures at baseline and after 6 months included SNOT‐20,^6^ Visual Analog Scale (VAS) for nasal and total symptoms, Asthma Control Test (ACT) score, pulmonary function testing (Jäger, Body, Würzburg), and oral corticosteroid dosage. All antibodies were prescribed by the treating pulmonologist solely on clinical grounds during routine care for indication of severe asthma, respecting EMA prescription criteria as well as administration and dosing according to the manufacturer's instructions. If the patient fulfilled prescription criteria for several antibodies, initial antibody choice was up to the physician's and patient's preferences. Statistical analyses were performed using GraphPad Prism 8 (GraphPad Software).

**TABLE 1:** Baseline characteristics of the study population according to treatment group. Data are displayed as mean +/− SD when normally distributed and otherwise median (IQR). Statistics by ordinary one‐way ANOVA or Kruskal‐Wallis ANOVA as appropriate

Baseline characteristics	Anti‐IgE (omalizumab)	Anti‐IL‐5/R (mepolizumab, benralizumab)	Anti‐IL‐4R (dupilumab)	*p*‐value (ANOVA)
Number of patients	9	26	15	
Age, mean (SD) years	56 (15.9)	60 (13.1)	51 (8.8)	**0.04**
Female, *n* (%)	8 (89)	12 (46)	5 (33)	
Age of onset, mean (SD) years	15 (6.8)	42 (16.8)	33 (14.3)	**0.0002**
BMI, mean (SD)	27.7 (6.4)	25.1 (4.2)	28.4 (4.6)	0.1
Previous antibody therapy, *n* (%)	1 (11)	13 (50)	11 (73)	
‐Herof previous therapy with:				
anti‐IgE	‐	3	0	
anti‐IL5/R	0	10*	11	
anti‐IL4R	1	0	0	
History of 2 previous antibodies	0	0	5	
Allergies, *n* (%)	9 (100)	17 (65)	7 (47)	
Season when antibody was initiated, *n* (%)				
Spring	6 (66)	13 (50)	3 (20)	
Summer	2 (22)	0 (0)	3 (20)	
Autumn	0 (0)	6 (23)	5 (33)	
Winter	1 (11)	7 (27)	4 (27)	
Polysensitization, *n* (%)	8 (89)	9 (35)	5 (33)	
Aspirin intolerance, *n* (%)	4 (44)	9 (35)	10 (67)	
Previous nasal polypectomy, *n* (%)	4 (44)	19 (76)	14 (93)	
OCS dependent patients – *n* (%)	3 (33)	13 (50)	2 (13)	
Prednisolone dose mg/d‐ median (range)	10 (7.5;10)	5 (2.5;70)	9 (8;10)	
Ann. Exacerbations before antibody‐median (range)	1.5 (0;3)	2 (0;12)	2 (0;12)	
Smoking history – *n* (%)				
Never	5 (56)	15 (58)	11 (73)	
Ex	4 (44)	11 (42)	4 (27)	
Active	0	0	0	
Packyears ex‐smokers‐ median (range)	7.5 (2.5;14)	8 (1;20)	7 (2;20)	
Symptom scores at baseline				
SNOT‐20, median (IQR)	55 (48–60)	52 (42–61)	56 (44–62)	0.76
ACT, median (IQR)	14 (11–19)	14 (10–20)	20 (10–23)	0.12
VAS – total symptoms, median (IQR)	4 (3–6)	5 (5–7)	8 (6–9)	**0.002**
VAS – nasal symptoms, median (IQR)	4 (0–7)	7 (5–9)	8 (5–9)	0.14
Lung function at baseline				
FEV1%, mean ± SD	59 ± 18	76 ± 24	81 ± 18	**0.047**
FVC%, mean ± SD	77 ± 8	92 ± 23	97 ± 18	0.056
FEV1/FVC%, mean ± SD	63 ± 17	66 ± 11	69 ± 9	0.57
MMEF%, mean ± SD	33 ± 38	42 ± 29	49 ± 21	0.44
RV/TLC%, mean ± SD	52 ± 14	45 ± 13	39 ± 10	**0.037**
Biomarkers at baseline				
Blood eosinophils at start of biological in cells/µl, median (IQR)	0.17 (0.06–0.34)	0.26 (0.04–0.74)	0.11 (0.00‐0.42)	0.31
highest historical blood eosinophils in cells/µl, median (IQR)	0.19 (0.13–0.38)	0.76 (0.52–1.36)	0.53 (0.42–0.83)	**0.0009**
IgE in IU/ml, median (IQR)	280 (54–700)	128 (47–455)	145 (80–348)	0.23
FeNO in ppb, median (IQR)	29 (15–43)	61 (36–102)	32 (22–69)	**0.018**

Abbreviations: ACT, asthma control test; ANOVA, analysis of variance; FEV1, forced expiratory volume in 1 sec; FVC, forced vital capacity; IQR, interquartile range; OCS, oral corticosteroids; SD, standard deviation; VAS, visual analoge scale.

*Switch anti‐IL5 to anti‐IL5R.

*p* < 0.05 was considered significant and marked in bold.

In total, 60 patients with severe asthma and nasal polyposis who initiated therapy between 2018 and 2020 were identified, hereof 10 patients were not included in the analysis due to following reasons: Five patients (two anti‐IgE and three anti‐IL5/R treated) were excluded due to missing baseline symptom scores (SNOT‐20 and VAS), three patients (all anti‐Il4R treated) had not reached 6 months timepoint yet at time of database closure. Two patients did not complete 6 months of treatment with the initiated antibody due to side‐effects: one patient stopped benralizumab after three injections due to headache reported repeatedly after applications; headaches did not reoccur after stopping therapy. Another patient stopped dupilumab after 3 months due occurrence hypereosinophilia and cough, received prednisolone and was switched to benralizumab which led to depletion of blood eosinophils and clearance of cough. No case of anaphylaxis was observed. Fifty patients were included in the efficacy analysis hereof treated with anti‐IgE (omalizumab): 9, anti‐IL‐5/R (mepolizumab/benralizumab): 26 and anti‐IL‐4R (dupilumab): 15 patients. Baseline characteristics of the anti‐IgE group differed from the other groups with a higher proportion of female patients, younger age of onset (*p* = 0.0002), higher percentage of allergies and lower FEV1 at baseline (*p* = 0.047; Table 1). Highest documented eosinophil count was significantly higher in patients treated with anti‐IL‐5/R and anti‐IL‐4R compared to the anti‐IgE group (*p* = 0.0009, Table 1). Moreover, patients treated with anti‐IgE rarely had previous antibody therapy and mostly started treatment in spring and summer, whereas history of previous antibody therapy was frequent in the other groups and treatment start was distributed among all seasons (Table 1). At baseline median SNOT‐20 was similar among groups (anti‐IgE: 55, anti‐IL‐5/R: 52 and anti‐IL‐4R: 56, *p* = 0.76), median visual analoge scale (VAS) for nasal symptoms was 4, 7 and 8 (*p* = 0.14) and VAS total symptoms was higher in the anti‐IL‐4R group (4, 5 and 8, *p* = 0.002, Table 1). Median baseline ACT score was 14, 14 and 20, respectively (*p* = 0.12, Table 1).

**FIGURE 1:**
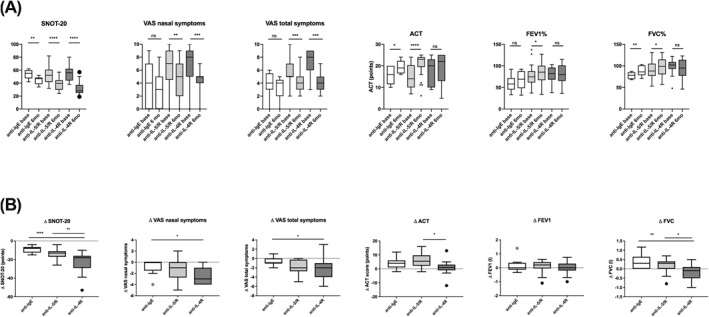
(A) Nasal and asthma outcomes at baseline and 6 months after treatment. German‐adapted version of Sino‐Nasal Outcome Test‐20 (SNOT‐20), nasal symptoms and total symptoms measured by visual analoge scale, Asthma Control Test, Forced Expiratory Volume in 1 s % predicted and Forced Vital Capacity % predicted were assessed before and after 6 months of anti‐IgE, anti‐IL‐5/R or anti‐IL‐4R‐therapy. Data shown as box and whisker plot (Tukey). Statistics: Wilcoxon matched‐pairs signed rank test; * <0.05, ** <0.01, *** <0.001, **** <0.0001. (B) Comparison of changes in outcome parameters after 6 months of treatment minus baseline values. All data are represented as box and whiskers (Tukey). Statistics: Kruskal‐Wallis + Dunn's multiple comparison test. * <0.05, ** <0.01, *** <0.001, **** <0.0001. For all displayed parameters in A + B: *n* (anti‐IgE) = 9, *n* (anti‐IL5/R) = 26, *n* (anti‐IL4R) = 15

After 6 months of antibody treatment, nasal symptoms measured by SNOT‐20 improved significantly in all patient groups with median improvement of anti‐IgE: −8 (*p* < 0.01), anti‐IL‐5/R: −13 (*p* < 0.001) and anti‐IL‐4R: −18 (*p* < 0.001, Figure 1A). VAS nasal symptoms improved by median anti‐IgE: 0 (n.s.), anti‐IL‐5/R: ‐1 (*p* < 0.01) and anti‐IL‐4R: ‐3 (*p* < 0.001), VAS total symptoms by‐1 (n.s.),‐2 (*p* < 0.001) and −2 (*p* < 0.001, Figure 1A). Asthma symptoms measured by ACT score improved compared to baseline by a median of +4 (*p* < 0.05), +5.5 (*p* < 0.001) and +1 points (n.s.). Pulmonary function testing improved numerically, with significant improvements found for FEV1% predicted (*p* = 0.01) in anti‐IL‐5/R group, and for FVC in anti‐IgE (*p* = 0.004) and anti‐IL‐5/R group (*p* = 0.025, Figure 1A). No nasal polyp operations were performed during the study period. One asthma exacerbation requiring OCS occurred in each the anti‐IgE and anti‐IL4R group and one CRSwNP exacerbation in the anti‐IL5/R group. Oral corticosteroid dose decreased significantly in the anti‐IL‐5/R group (*n* = 13, median difference = −3 mg, *p* = 0.0049) hereof four completely stopped OCS. The numbers of patients receiving regular OCS at start of antibody was small in anti‐IgE (*n* = 3) and anti‐IL‐4R (*n* = 2) group. Comparing changes during therapy (Δ = values at 6 months minus baseline values) between the different treatment groups, larger improvement of SNOT‐20 (ΔSNOT‐20) was observed in the anti‐IL‐4R group than in anti‐IgE (*p* < 0.001) and anti‐IL‐5/R (*p* < 0.001) groups (Figure 1B). ΔVAS nasal and total symptoms improvement was significantly higher in the anti‐IL‐4R group than in the anti‐IgE group (*p* < 0.05). Increase in ACT was higher in anti‐IL‐5/R compared to anti‐IL‐4R treated patients (*p* < 0.05) and increase in FVC was higher in anti‐IgE treated patients compared to the other groups (anti‐IgE vs. anti‐IL‐5/R: *p* < 0.05, anti‐IgE vs. anti‐IL‐4R: *p* < 0.01, Figure 1B).

We found that treatment with anti‐IgE, anti‐IL‐5/R and anti‐IL‐4R while improving asthma outcomes, also all significantly reduced the symptoms of CRSwNP measured by SNOT‐20, supporting the “one airway concept” that assumes similar pathomechanisms in CRSwNP and asthma.^7^ Recent trials in severe CRSwNP have shown effectiveness of dupilumab,^2^ omalizumab^3^ and mepolizumab^4^ reporting improvements in objective outcome measures like endoscopic nasal polyp score (NPS) as well as subjective measures, including SNOT‐22. In our study, the different treatment groups varied in baseline characteristics reflecting the different target molecules and licensing criteria of the antibodies in severe asthma and this limits comparability between groups. Thus, the dominant phenotype was severe allergic asthma with typically early‐onset disease in anti‐IgE‐treated patients, late‐onset eosinophilic asthma in anti‐IL‐5/R‐treated patients and somewhat mixed phenotype with eosinophilia in anti‐IL‐4R‐treated patients. Moreover, baseline nasal symptoms were lower at baseline in the anti‐IgE group, leaving less room for improvement than in the other groups. Whether patients with allergen‐driven asthma are generally less likely to have severe rhinosinusitis than those with non‐allergic phenotype needs to be addressed in larger studies.

With different targeted treatments for severe asthma at hand and many patients fulfilling indication criteria for several antibodies, pulmonologists have asked the question whether the presence of comorbid nasal polyps represents a phenotypic feature favoring one targeted treatment over the other. Yet, it can also be hypothesized that the phenotype of CRSwNP of an individual patient is similar to his asthma phenotype and hence choosing according to asthma phenotype would also fit for CRSwNP. While these questions cannot be definitely answered without comparative RCTs, our study provides some indications for both hypotheses: on the one hand, all patient groups showed improvement of nasal symptoms with the antibody given according to the asthma indication. On the other hand, dupilumab treated patients had the greatest benefit while being pretreated with another antibody most frequently. Thus, asthmatic patients who still have high symptom burden of CRSwNP comorbidity under antibody treatment might benefit from a switch to dupilumab. Limitations of the study include retrospective design, limited sample size, and baseline differences between treatment groups. Moreover, as treatment effects were observed over a 6 months‐period only, seasonal effects cannot be excluded.

In sum, treatment by all studied antibodies showed real‐life effectiveness in reducing symptoms of nasal polyps in patients with severe asthma. Differences in treatment effects with larger benefit on nasal outcomes in anti‐IL4R‐treated patients were found and should be regarded as hypothesis generating for future studies.

## CONFLICTS OF INTEREST

Carlo Mümmler, Kristin Dünzelmann, Michaela Barnikel, Dieter Munker, Martin Canis: declare no conflicts of interest. Katrin Milger: reports speaker fees for Astrazeneca, GSK, Novartis, Sanofi. Andrea Koch: reports speaker fees for AstraZeneca, Novartis, Sanofi. Frank Haubner: reports speaker fees for Brainlab, Spiggle&Theis. Jürgen Behr: reports speaker fees from AstraZeneca and Novartis. Nikolaus Kneidinger: reports speaker fees from Astrazenca and Sanofi. Moritz Gröger: reports speaker fees from Novartis and Sanofi.

## AUTHOR CONTRIBUTIONS

Perception of the study: Andrea Koch, Frank Haubner and Katrin Milger. Data collection and patient treatment: Carlo Mümmler, Kristin Dünzelmann, Nikolaus Kneidinger, Michaela Barnikel, Dieter Munker, Moritz Gröger, Martin Canis, Jürgen Behr, Frank Haubner. Data analysis and manuscript draft: Carlo Mümmler, Kristin Dünzelmann and Katrin Milger. Manuscript revision and approval: all authors.
